# Supplementary material for: Somatic mutation phasing and haplotype extension using linked-reads in multiple myeloma
Source: bioRxiv. 2024 Aug 10:2024.08.09.607342. Preprint. [Version 1] doi: 10.1101/2024.08.09.607342 (PMC11326269; doi:10.1101/2024.08.09.607342)
Supplement: Supplement 2 [file NIHPP2024.08.09.607342v1-supplement-2.pdf]

bioRxiv preprint doi: https://doi.org/10.1101/2024.08.09.607342; this version posted August 10, 2024. The copyright holder for this preprint (which was not certified by peer review) is the author/funder, who has granted bioRxiv a license to display the preprint in perpetuity. It is made available under aCC-BY 4.0 International license.

Supplementary Figure Legends

Supplementary Figure 1. Phasing performance quality control summary measures for our tumor and normal samples plus 1000 Genomes samples NA12878 (+) and NA19240 (x). Violin plots defined as: center line, median; violin limits, minimum and maximum values; points, every observation. Definitions of metrics may be found here: https://support.10xgenomics.com/genome-exome/software/pipelines/latest/output/metrics.

Supplementary Figure 2. Phase block length distribution. a. Phase block length by chromosome across all samples. Outlier phase blocks from sample 25183 (Rel) circled. Violin plots defined as: center line, median; violin limits, minimum and maximum values; points, every observation. **b.** Phase block length per sample across all chromosomes. **c.** Phase block lengths of chr13, chr22, and others from 27522 (P). Phase blocks less than 1 kb filtered out for plotting. **d.** Chr13 and chr22 phase block boundaries from 27522 (P) and 27522 (Rem). Alternating dark and light boxes indicate adjacent phase blocks. **e.** Total phase block genome coverage from all samples combined, grouped by phase block length.

Supplementary Figure 3. Copy number profile of Patient 27522 at the primary disease stage. Y-axis values are copy number ratios on the log2 scale.

Supplementary Figure 4. Additional information related to somatic mutation phasing. **a.** Precision/recall rates at various cutoffs for the proportion of linked-alleles assigned to one haplotype. **b.** Comparison of phasing results with Long Ranger genotypes.

Supplementary Figure 5. Additional information related to the relationship of pairs of somatic mutation. **a.** Number of barcodes covering each mutation site and those supporting the mutant allele. **b.** Number of overlapping barcodes by distance between somatic mutations less than 100 bp apart.

Supplementary Figure 6. Barcodes supporting 27522 (P) NRAS hotspot mutation pair.

bioRxiv preprint doi: https://doi.org/10.1101/2024.08.09.607342; this version posted August 10, 2024. The copyright holder for this preprint (which was not certified by peer review) is the author/funder, who has granted bioRxiv a license to display the preprint in perpetuity. It is made available under aCC-BY 4.0 International license.

Supplementary Figure 7. Common myeloma translocations mapped to haplotypes. a. Overlap of translocations observed in 27522 (P) and (Rel). **b.** Model of t(4;14) translocation. **c.** Barcodes supporting t(4;14) indicate a single haplotype origin. **d.** Translocations observed in 77570 (P). **e.** Model of t(11;14) translocation. **f.** Barcodes supporting t(11;14) indicate a single complex event.

Supplementary Figure 8. Barcode support for common myeloma translocations. **a-b.** 27522 (P) t(4;14). **c-f.** 77570 (P) t(11;14).

bioRxiv preprint doi: https://doi.org/10.1101/2024.08.09.607342; this version posted August 10, 2024. The copyright holder for this preprint (which was not certified by peer review) is the author/funder, who has granted bioRxiv a license to display the preprint in perpetuity. It is made available under aCC-BY 4.0 International license.

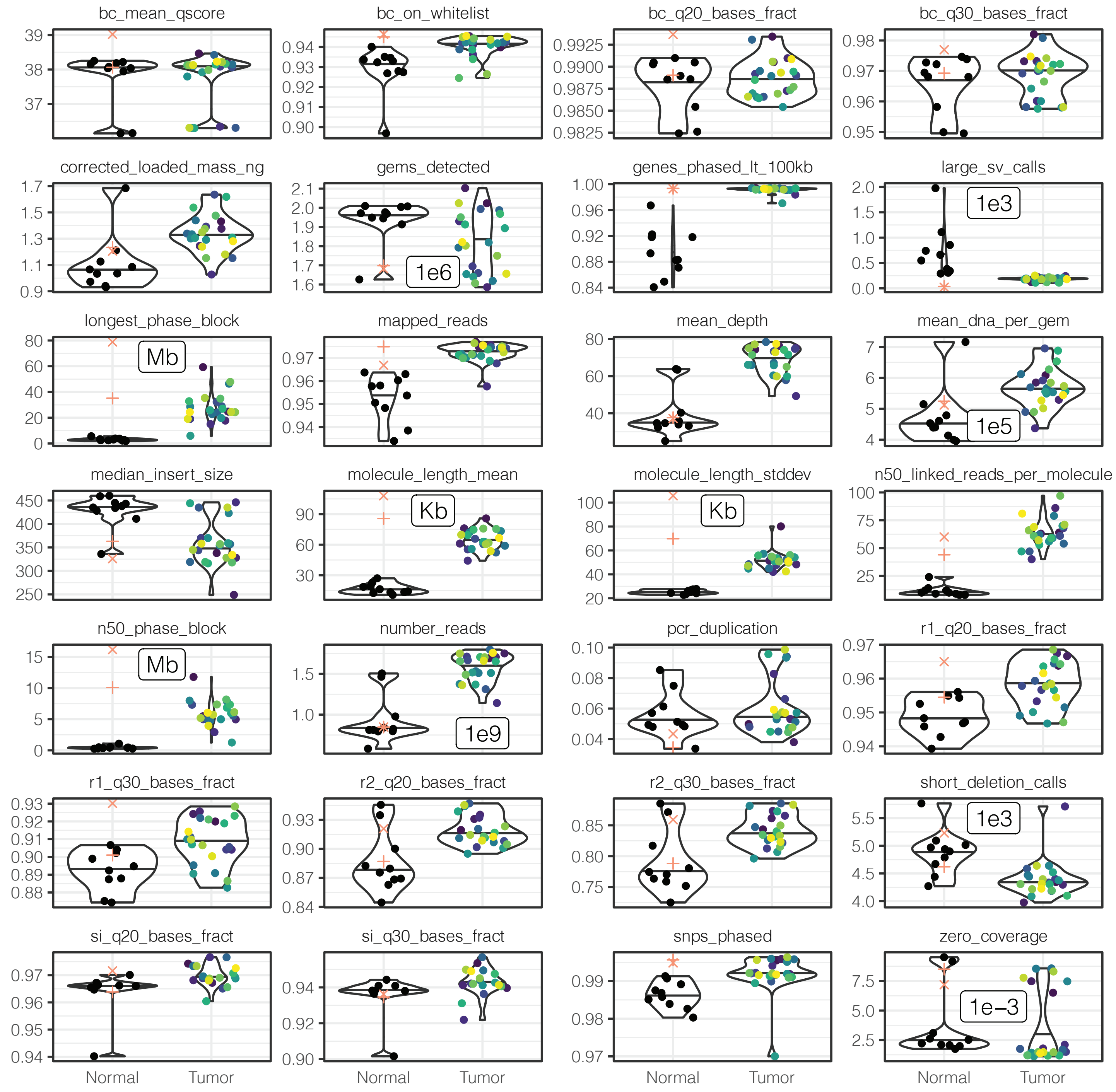

Supplementary Figure 1. Phasing performance quality control summary measures for our tumor and normal samples plus 1000 Genomes samples NA12878 (+) and NA19240 (x). Violin plots defined as: center line, median; violin limits, minimum and maximum values; points, every observation. Definitions of metrics may be found here: https://support.10xgenomics.com/genome-exome/software/pipelines/latest/output/metrics.

bioRxiv preprint doi: https://doi.org/10.1101/2024.08.09.607342; this version posted August 10, 2024. The copyright holder for this preprint (which was not certified by peer review) is the author/funder, who has granted bioRxiv a license to display the preprint in perpetuity. It is made available under aCC-BY 4.0 International license.

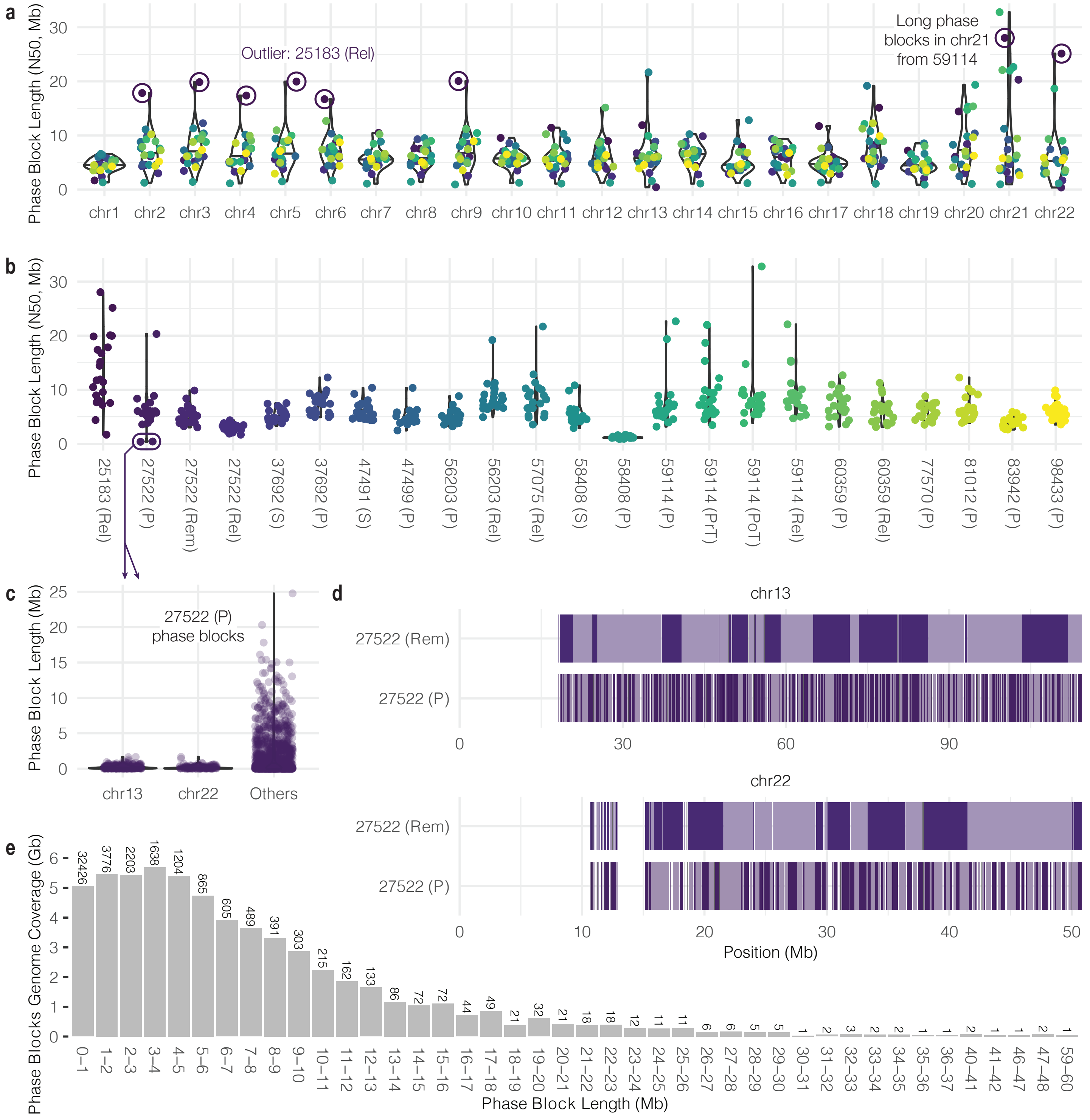

Supplementary Figure 2. Phase block length distribution. a. Phase block length by chromosome across all samples. Outlier phase blocks from sample 25183 (Rel) circled. Violin plots defined as: center line, median; violin limits, minimum and maximum values; points, every observation. b. Phase block length per sample across all chromosomes. c. Phase block lengths of chr13, chr22, and others from 27522 (P). Phase blocks less than 1 kb filtered out for plotting. d. Chr13 and chr22 phase block boundaries from 27522 (P) and 27522 (Rem). Alternating dark and light boxes indicate adjacent phase blocks. e. Total phase block genome coverage from all samples combined, grouped by phase block length.

bioRxiv preprint doi: https://doi.org/10.1101/2024.08.09.607342; this version posted August 10, 2024. The copyright holder for this preprint (which was not certified by peer review) is the author/funder, who has granted bioRxiv a license to display the preprint in perpetuity. It is made available under aCC-BY 4.0 International license.

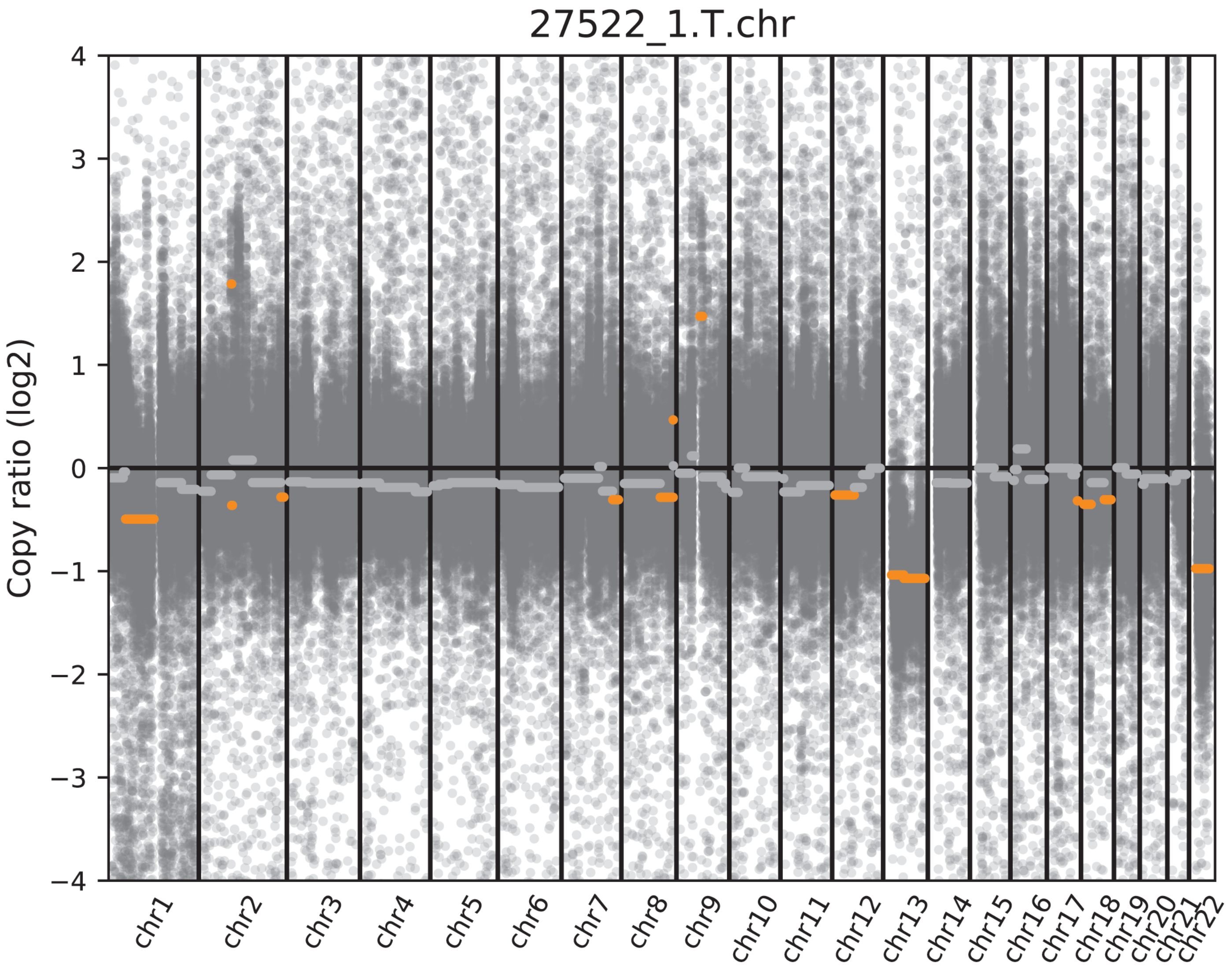

Supplementary Figure 3. Copy number profile of Patient 27522 at the primary disease stage. Y-axis values are copy number ratios on the log2 scale.

bioRxiv preprint doi: https://doi.org/10.1101/2024.08.09.607342; this version posted August 10, 2024. The copyright holder for this preprint (which was not certified by peer review) is the author/funder, who has granted bioRxiv a license to display the preprint in perpetuity. It is made available under aCC-BY 4.0 International license.

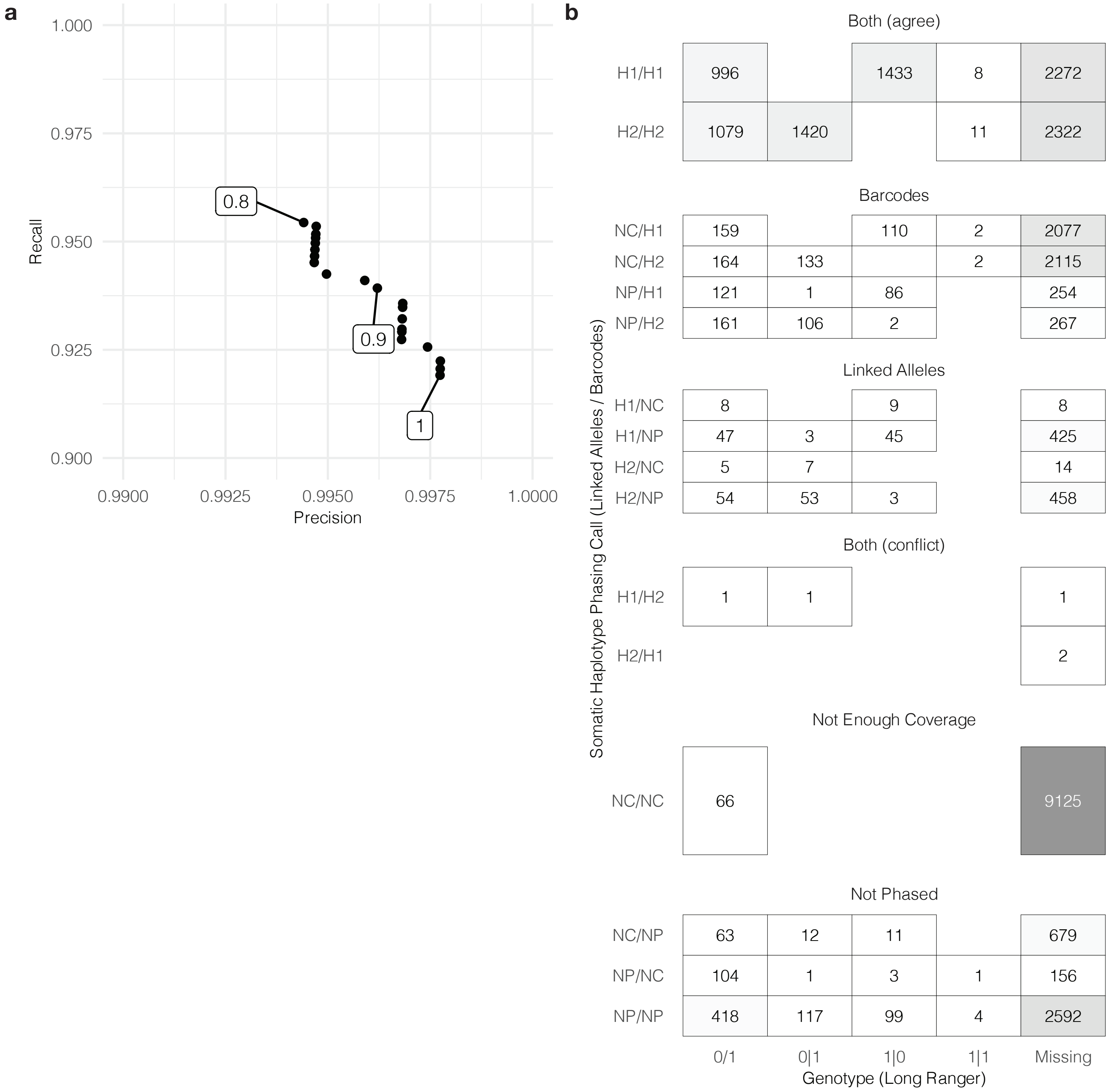

Group	Call	0/1	0|1	1|0	1|1	Missing
Both (agree)	H1/H1	996		1433	8	2272
Both (agree)	H2/H2	1079	1420		11	2322
Barcodes	NC/H1	159		110	2	2077
Barcodes	NC/H2	164	133		2	2115
Barcodes	NP/H1	121	1	86		254
Barcodes	NP/H2	161	106	2		267
Linked Alleles	H1/NC	8		9		8
Linked Alleles	H1/NP	47	3	45		425
Linked Alleles	H2/NC	5	7			14
Linked Alleles	H2/NP	54	53	3		458
Both (conflict)	H1/H2	1	1			1
Both (conflict)	H2/H1					2
Not Enough Coverage	NC/NC	66				9125
Not Phased	NC/NP	63	12	11		679
Not Phased	NP/NC	104	1	3	1	156
Not Phased	NP/NP	418	117	99	4	2592

Supplementary Figure 4. Additional information related to somatic mutation phasing. a. Precision/recall rates at various cutoffs for the proportion of linked-alleles assigned to one haplotype. b. Comparison of phasing results with Long Ranger genotypes.

bioRxiv preprint doi: https://doi.org/10.1101/2024.08.09.607342; this version posted August 10, 2024. The copyright holder for this preprint (which was not certified by peer review) is the author/funder, who has granted bioRxiv a license to display the preprint in perpetuity. It is made available under aCC-BY 4.0 International license.

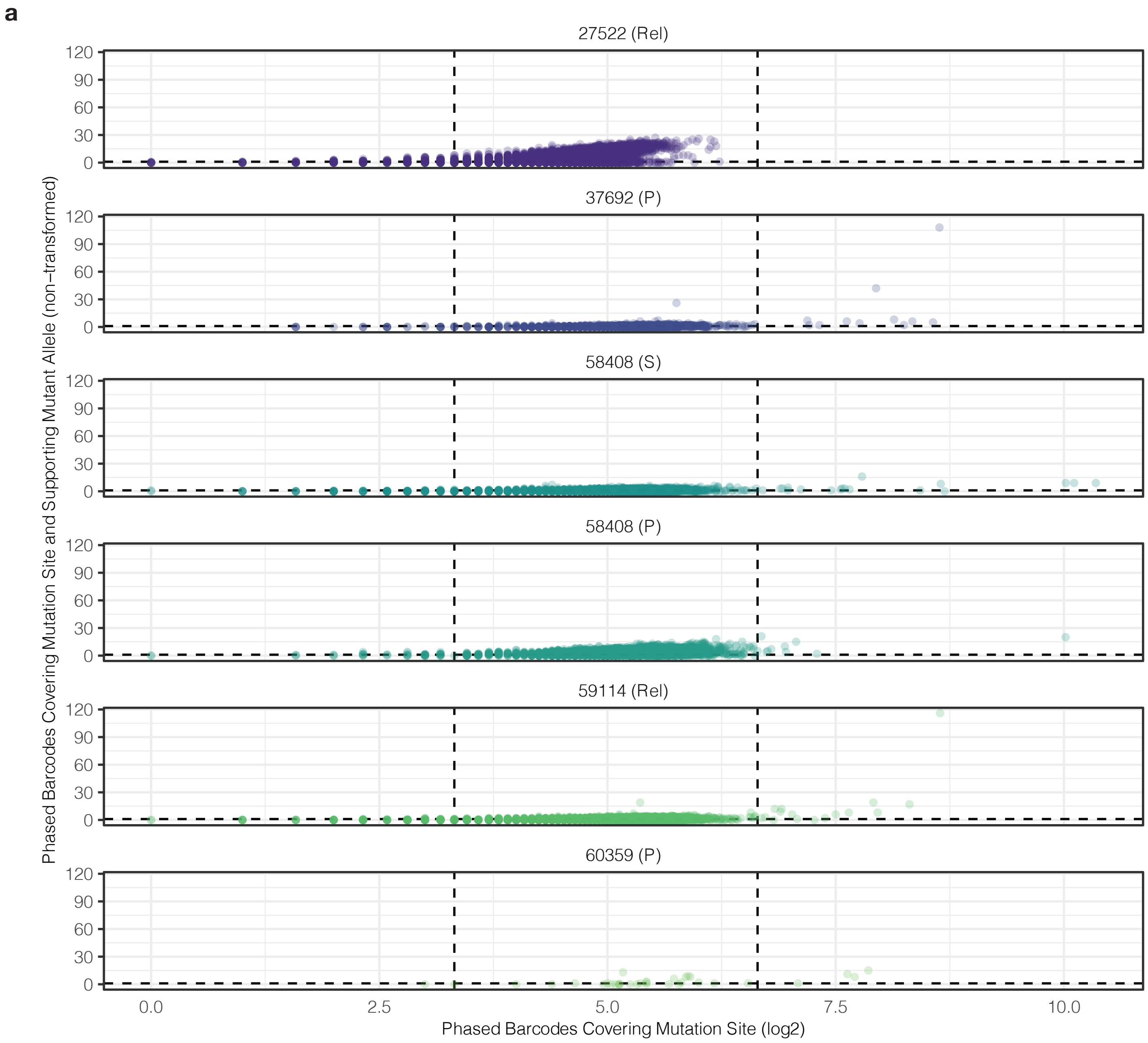

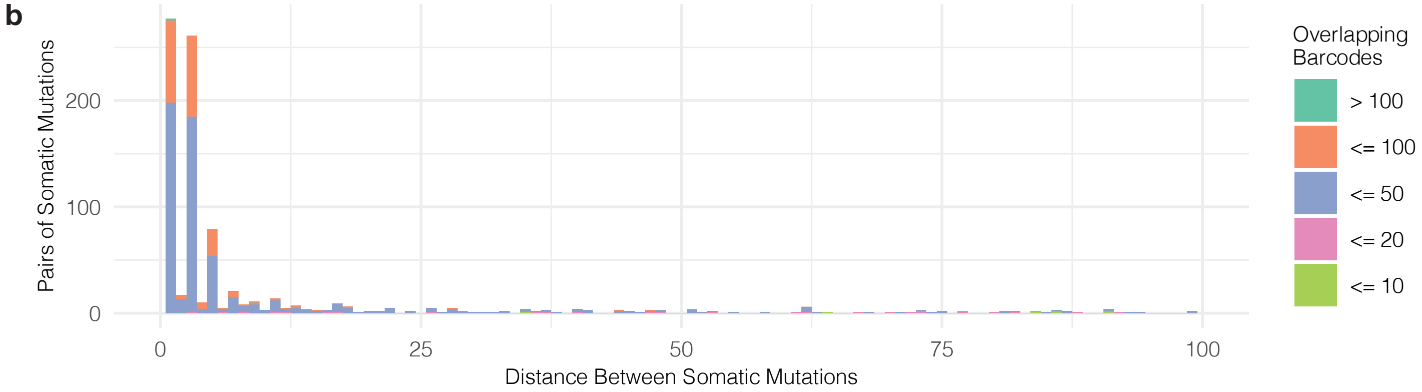

Supplementary Figure 5. Additional information related to the relationship of pairs of somatic mutation. a. Number of barcodes covering each mutation site and those supporting the mutant allele. b. Number of overlapping barcodes by distance between somatic mutations less than 100 bp apart.

bioRxiv preprint doi: https://doi.org/10.1101/2024.08.09.607342; this version posted August 10, 2024. The copyright holder for this preprint (which was not certified by peer review) is the author/funder, who has granted bioRxiv a license to display the preprint in perpetuity. It is made available under aCC-BY 4.0 International license.

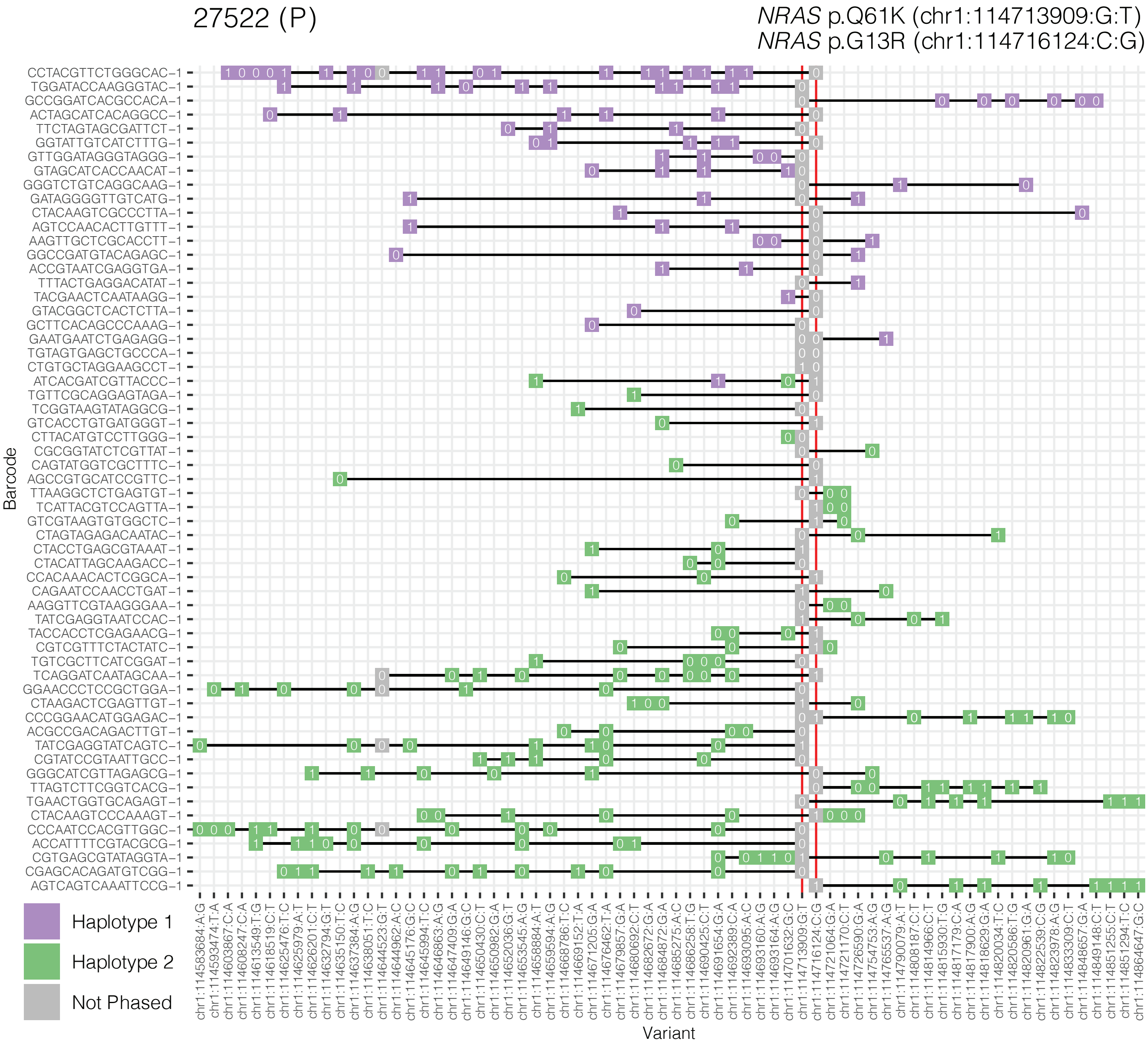

Supplementary Figure 6. Barcodes supporting 27522 (P) NRAS hotspot mutation pair.

bioRxiv preprint doi: https://doi.org/10.1101/2024.08.09.607342; this version posted August 10, 2024. The copyright holder for this preprint (which was not certified by peer review) is the author/funder, who has granted bioRxiv a license to display the preprint in perpetuity. It is made available under aCC-BY 4.0 International license.

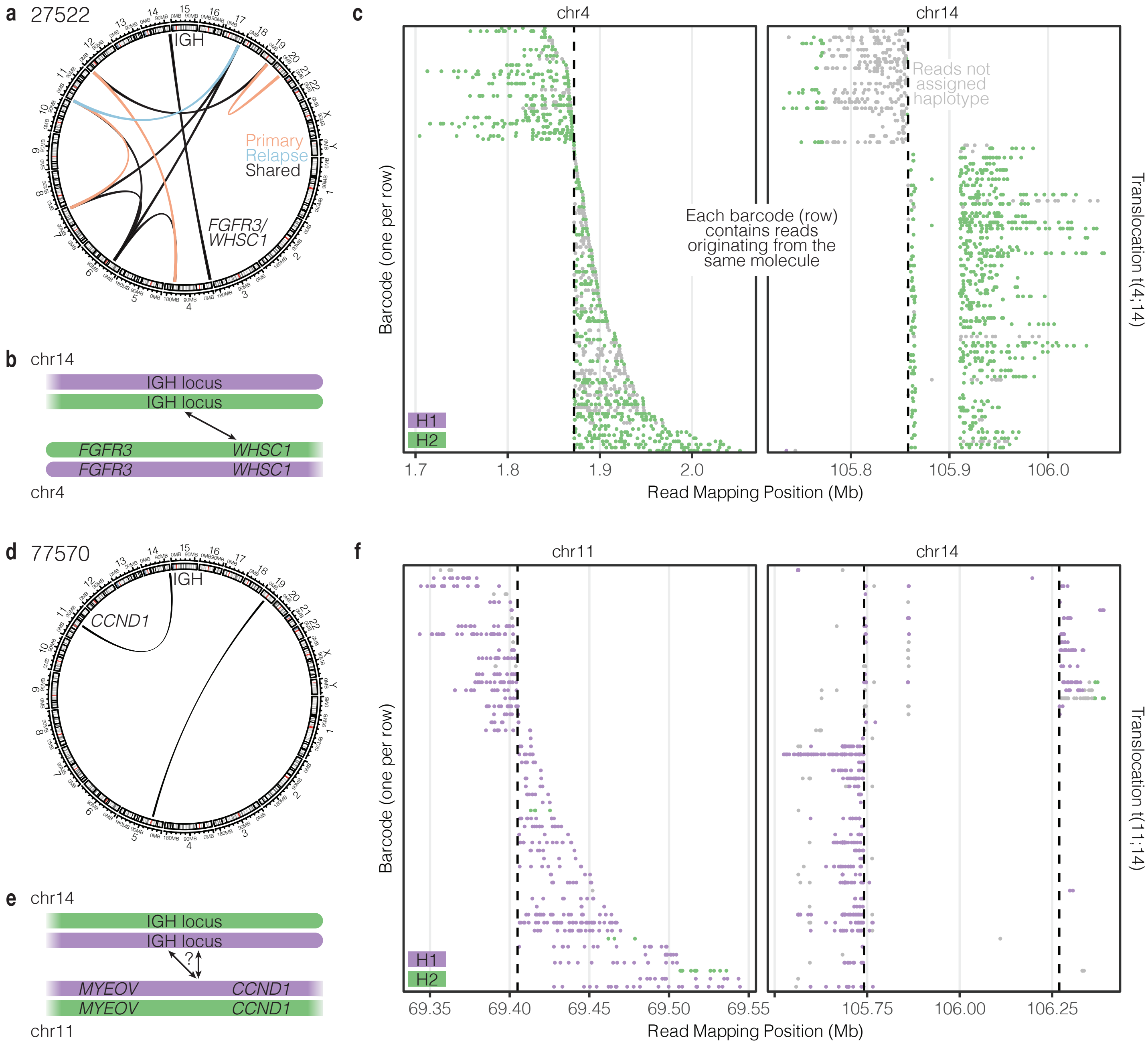

Supplementary Figure 7. Common myeloma translocations mapped to haplotypes. a. Overlap of translocations observed in 27522 (P) and (Rel). b. Model of t(4;14) translocation. c. Barcodes supporting t(4;14) indicate a single haplotype origin. d. Translocations observed in 77570 (P). e. Model of t(11;14) translocation. f. Barcodes supporting t(11;14) indicate a single complex event.

bioRxiv preprint doi: https://doi.org/10.1101/2024.08.09.607342; this version posted August 10, 2024. The copyright holder for this preprint (which was not certified by peer review) is the author/funder, who has granted bioRxiv a license to display the preprint in perpetuity. It is made available under aCC-BY 4.0 International license.

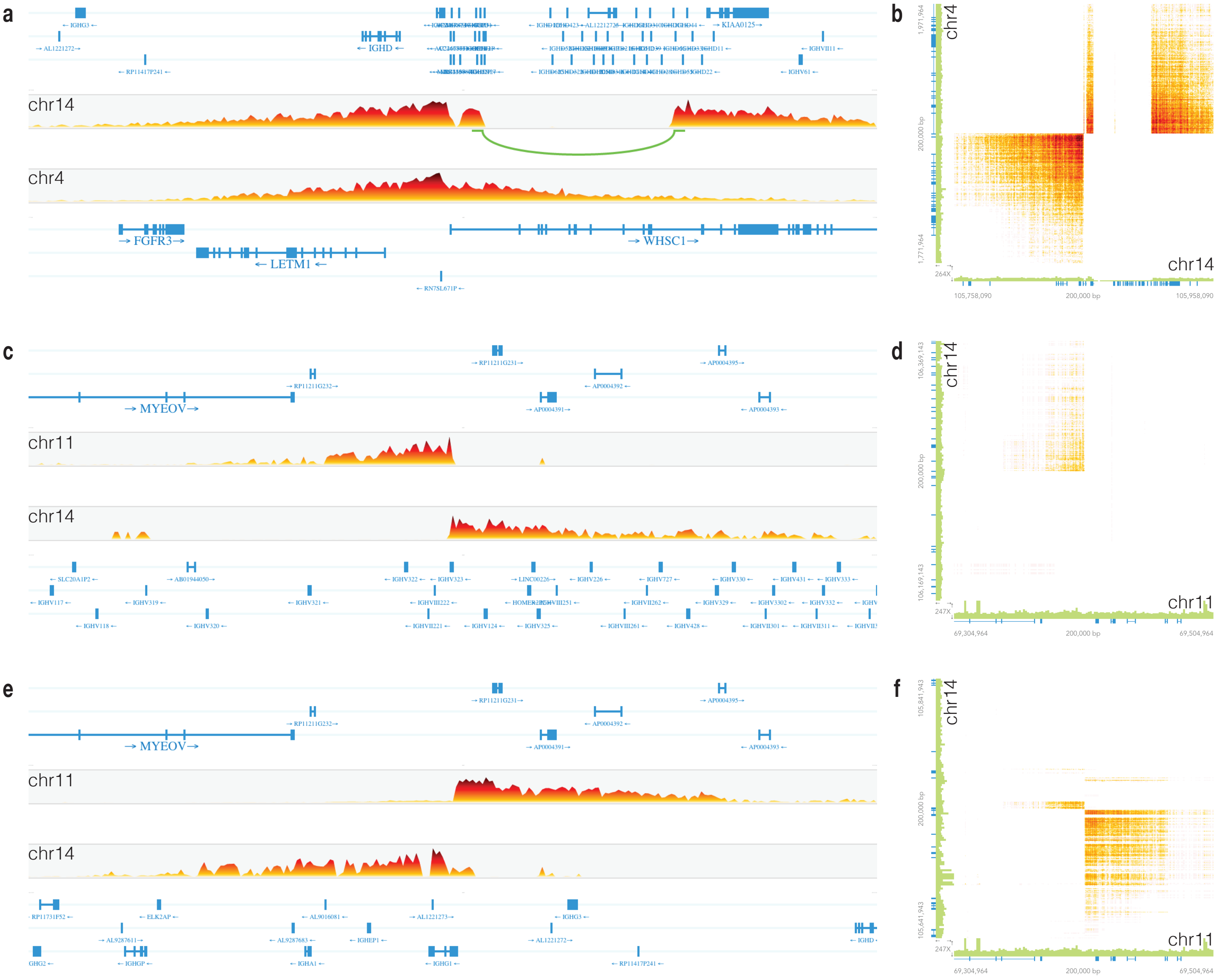

Supplementary Figure 8. Barcode support for common myeloma translocations. a-b. 27522 (P) t(4;14). c-f. 77570 (P) t(11;14).
